# Omnidirectional Electric Fish

**DOI:** 10.1371/journal.pbio.0050314

**Published:** 2007-11-13

**Authors:** Kira E O'Day

What could an African lion stalking a distant impala in the African savanna possibly have in common with an electric fish searching for nearby water fleas in the Amazon River? Both the lion and the rather less daunting 14-cm black ghost knifefish (*Apteronotus albifrons*) use sensory information to help them to locate and catch prey. But lions, which rely on their acute vision during the hunt, engage in passive sensing, whereas the knifefish actively senses by generating a weak electric field around its body. And while lions and most other predators both sense and move mainly in the forward direction, the knifefish can sense in all directions and swim rapidly backward and forward to intercept its prey.

Although it's certainly useful to be able to sense in all directions, active sensing comes at a cost; energetically, it's very expensive to generate a good-sized electric field, since the signal falls off rapidly with distance. To see whether the energetic constraints of the knifefish's active sensing might lead to a restricted sensory space when compared with passive-sensing creatures like lions, and to study the relationship between the knifefish's sensory capability and its extraordinary movements while hunting for food, Malcolm MacIver along with graduate student James Snyder and colleagues measured the sensory volume (*SV*)—the size and shape of the space within which objects can be detected by an animal—and compared it to the motor volume (*MV*)—the location in space that an animal can reach within a set time period by activating its musculoskeletal system.

To do this, the researchers computationally modeled the knifefish's *SV* using measurements and models of the prey (water fleas [*Daphnia magna*]), the fish's electric field, the distribution of and activation of the sensory receptors arranged along the fish's body, and its behavior during capture of the water fleas. To measure *MV*, the authors turned to a previous study, which examined prey capture behavior by introducing individual water fleas into a knifefish enclosure and videotaping the fish as it sensed the presence of prey and moved in for the kill. The knifefish, when searching for prey, swims forward at a speed of not quite 10 cm/s, with its head pitched downward at a 30° angle. When the knifefish detects food, a ribbon fin running along its back allows it to abruptly decelerate and change direction to intercept its prey. Data gleaned from 116 prey-capture trials were used by Snyder et al. to compute the *MV* in all directions.

**Figure:**
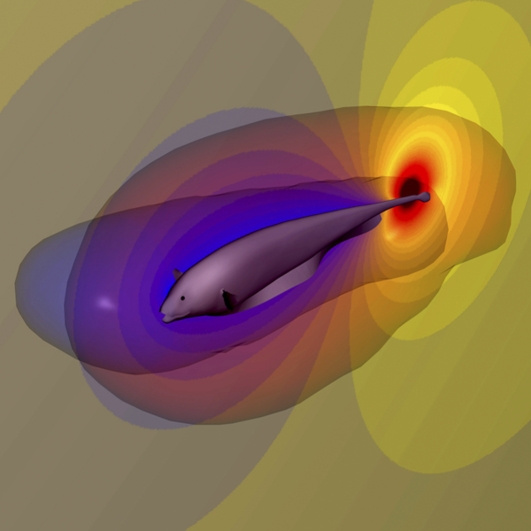
A computer model of the knifefish illustrates the estimated *SV* for active sensing of prey (red) and stopping *MV* (blue). The backdrop shows a color map of the fish's simulated self-generated electric field. *SV* barely exceeds the stopping *MV*, revealing that the fish invests just enough energy into active sensing to detect prey in time to stop.

The researchers found that there is a close connection between the size and shape of the *SV* and *MV*, which roughly overlap. On average, the estimated prey detection distance is about 3.5 cm from the fish's body. Both the *SV* and *MV* are omnidirectional and cylindrical in shape. By reanalyzing the results of a previous study that measured prey capture behavior in water with differing electrical conductivities (which influence the distance at which prey can be detected), the authors were also able to determine that when the conductivity of the water increased, leading to a decrease in the *SV*, the knifefish decreased the speed at which it swam while looking for food, correspondingly decreasing the size of the *MV*. This indicates that the knifefish can make behavioral adjustments to maintain the match between the *SV* and *MV*, helping it to avoid colliding with objects lying outside of its electrical field—a significant benefit in the murky, cluttered rivers of the Amazon Basin.

Snyder et al. then compared the overlapping relationship between the *SV* and *MV* in knifefish with that of other animals that use a variety of sensing mechanisms. For active-sensing bats, which emit bursts of ultrasonic energy into the environment to detect prey, the *SV* is in a cone shape extending forward from its head. Although the bat *MV* hasn't been determined yet, based on known parameters such as cruising speed and deceleration, its predicted *MV* appears roughly comparable to its *SV*. This isn't the case with dolphins, which also actively sense using ultrasonic energy. Dolphins can sense prey up to 100 m away, well beyond the range of their predicted *MV* and more on par with the *SV*:*MV* relationship of a passive-sensing animal like the lion, which can also sense prey far beyond its immediate reach using a combination of sight and sound.

Although the knifefish is singular in that its *SV* and *MV* are omnidirectional, the findings from this study suggest that measuring *SV* and *MV* will be quite useful for studying predator–prey strategies, not only in active-sensing animals like electric fish, bats, and dolphins, but also in more common passive-sensing animals, which rely on vision and hearing. For example, when the *SV*:*MV* ratio is close to 1, fast reactive strategies are favored, while more complex, longer-range planning strategies are possible as the relative size of the *SV* increases. Measuring the *SV* and *MV* of animals in a variety of contexts may serve to highlight important and as-yet undiscovered functional relationships between sensing, movement, and behavior.

